# Clinical and Biochemical Features in a Case of Familial Hypocalciuric Hypercalcemia Type 3 with *AP2S1* Gene Mutation in Codon Arg15His

**DOI:** 10.1155/2020/7312894

**Published:** 2020-01-28

**Authors:** Mohamed Aashiq, Asma Jassim Malallah, Farheen Khan, Maryam Alsada

**Affiliations:** Department of Pediatrics, Dubai Hospital, Dubai Health Authority, Dubai, UAE

## Abstract

Familial hypocalciuric hypercalcemia (FHH) is usually a benign condition divided into three types. FHH-3 occurs in about 20% of the cases and is caused due to missense mutations in *AP2S1* (adaptor-related protein complex 2 subunit sigma 1) involving the codon Arg15 (p.R15). We report a case of FHH-3 with a heterozygous mutation in the *AP2S1* gene on chr19_47349359 C>T, c.44G>A, p.Arg15His. There are a handful of reports describing the clinical features in patients diagnosed with FHH-3. Herein, we describe the laboratory and clinical features associated with a case of FHH-3 with mutation in the Arg15His codon of the *AP2S1* gene.

## 1. Introduction

Familial hypocalciuric hypercalcemia (FHH) is a benign autosomal dominant condition, characterized by an increased serum calcium and inappropriately low urinary calcium with normal serum parathyroid hormone (PTH) values [1]. FHH can be classified into three types based on chromosomal mutational hotspots at 3q13.3–21, 19p13.3, and 19q13.3 [2–5]. FHH-1, which constitutes approximately 65% of FHH, results from inactivating mutation in a gene encoding for calcium-sensing receptor (CaSR), a G-protein-coupled receptor encoded by the *CASR* gene on chromosome 3q13.3–21 [4, 6]. FHH-2 is caused by inactivating mutations in the *GNA11* gene encoding G-protein subunit *α*_11_ on chromosome 19p13.3 [2].

FHH-3 is the result of missense mutations on *AP2S1*, involving the Arg15 residue (Arg15Cys, Arg15His, and Arg15Leu), and is a cause in 20% of cases of FHH [5, 7]. The AP2S1 complex is important for clathrin-coated vesicle- (CCV-) mediated endocytosis; mutations in the *AP2S1* gene encoding the sigma subunit of the adaptor protein 2 (AP2) complex inhibits this mechanism [5, 8], thereby decreasing the calcium sensitivity of cells expressing CaSR protein to extracellular calcium, which regulates PTH secretion from parathyroid glands and tubular reabsorption of calcium in the kidneys, resulting in disordered calcium homeostasis [2, 6, 9].

We report a case of FHH-3 with *AP2S1* gene mutation in the Arg15His codon, enlisting the biochemical features seen in the proband and his mother along with the clinical features in the proband.

## 2. Case Report

A 4-year-old boy of Arab origin was first evaluated for hypercalcemia at the age of 9 months when he presented with an episode of febrile convulsion. His routine laboratory tests at the time of presentation showed an elevated calcium level. His parents are nonconsanguineous. He is a late preterm child, delivered by normal vaginal delivery at a gestational age of 35 weeks. He had no significant postnatal events and was discharged home after birth. His surgical history is significant for repair of bilateral inguinal hernia. There is a family history of congenital heart disease in the mother in the form of pulmonary stenosis treated with balloon dilatation and a sister with PDA (patent ductus arteriosus) closed by catheterization. His mother has alpha-thalassemia trait, and his father had bilateral congenital cataract.

On admission and further evaluation for hypercalcemia, he was found to have persistent hypocalciuria (decreased urinary calcium to creatinine ratio adjusted for age) and slightly elevated magnesium levels, with normal PTH, phosphate, and vitamin D levels and high alkaline phosphatase. On evaluation of the mother, she was also found to have hypercalcemia, hypocalciuria with normal magnesium, phosphate, and alkaline phosphatase, and low vitamin D levels (Table 1).

On examination, the proband had subtle facial dysmorphic features in the form of upslanting palpebral fissures, wide mouth, and high-arched palate with normal growth parameters. A complete evaluation of the patient including karyotype analysis, FISH (fluorescence in situ hybridization) for Williams syndrome, renal USS (ultrasound scan), brain CT (computed tomography), and ECHO (Echocardiography) were all normal.

Considering the laboratory results, familial hypocalciuric hypercalcemia was suspected. A molecular genetic analysis of the calcium-sensing receptor gene (*CASR*) was done for the 6 coding exons (exons 2–7) and the exon-intron boundaries of the *CASR* gene on chromosome 3q21.1, which were amplified by polymerase chain reaction (PCR) and sequenced directly. On comparing the resulting sequence data with the reference sequence NM_001178065.1, no change of pathogenic relevance was detected. Following this, a deletion/duplication analysis of the *CASR* gene was performed by applying MLPA (multiple ligation-dependent probe amplification) using the SALSA kit P177–B1. Exons 1–8 (NM_000388.3) were screened for deletions or duplications and were found to be normal.

Following the negative results for *CASR* gene analysis, a whole exome sequencing (WES) was done for both the proband and his parents. WES by Sanger sequencing confirmed a heterozygous mutation in the *AP2S1* gene, on chr19_47349359 C>T, c.44G>A, p.Arg15His in both the proband and his mother.

On further follow-up in the clinic since first being suspected with FHH, he was found to have developmental and speech delay with language skill deficit. Brain MRI (magnetic resonance imaging) was performed for the evaluation of developmental delay and showed no focal lesions in the brain parenchyma. The proband was also diagnosed with ADHD (attention deficit hyperactivity disorder) at the age of 3 years. Currently, the child is otherwise thriving well and is not on any treatment for hypercalcemia except for regular follow-up since the calcium levels were only mildly elevated. He is regularly followed up in the pediatric endocrine, neurology, and occupational and speech therapy clinics.

## 3. Discussion

We confirmed the diagnosis of FHH Type 3 in our proband with WES at the age of 2 years 8 months, and we had followed him regularly and had him evaluated for his ADHD features and developmental and speech delay. We have reported his laboratory investigations and clinical features to further add to the reports, showing FHH-3 and its association with cognitive impairment [10–12].

The child in our case, along with his mother, has a heterozygous mutation in the codon Arg15His (p.R15H) of the *AP2S1* gene. To our knowledge, this is the first reported case of FHH-3 in the region and the ninth reported case of Arg15His residue mutation in the literature.

Since establishing *AP2S1* gene mutation as a cause of FHH-3, a few reports have been published to determine the differences in phenotypes between FHH-3 and classical FHH. A couple of reports by Nesbit et al. showed no differences in features with patients having FHH-3 and classical FHH [5, 7]. Hannan et al. in their series revealed phenotypic differences between individuals with FHH-3 and FHH-1, which were similar to the findings reported by Vargas-Poussou et al. that individuals with FHH-3 had higher plasma calcium and an increased renal tubular reabsorption of calcium [11, 13].

Hendy et al. reported three patients with *AP2S1* gene mutation: two cases with Arg15Leu and Arg15Cys mutations had major depression and an undescribed psychiatric condition, respectively, while the third patient with Arg15Leu had cerebral palsy and global developmental delay [10]. Hannan et al. in their series noted that cases with Arg15Leu mutation had marked hypercalcemia, whereas Arg15His mutations were associated with mild increase in serum calcium similar to our case. In addition, they reported that individuals with FHH-3 were more likely to have cognitive impairment when compared with individuals with FHH-1, raising the possibility of an association between *AP2S1* mutation and cognitive impairment [11]. This possibility was also highlighted by Szalat et al. in their series, where two of their patients with FHH-3 had cognitive disorder, depression, severe ADHD, and language skill deficiency [12].

AP2 plays an important role in clathrin-mediated endocytosis, which is important for membrane protein trafficking and neurotransmission [14]. Clathrin-mediated endocytosis has been hypothesized to be involved in abnormal neurodevelopment and psychotic disorders like bipolar and schizophrenia [15]. AP2 has also been found to mediate AMPA(α-amino-3-hydroxy-5-methyl-4-isoxazolepropionic acid) receptor trafficking in the hippocampus which is associated with behavior and depression [16, 17]. *AP2S1* gene mutation in Arg15 has been shown to inhibit the clathrin-coated vesicular transport resulting in features of FHH [5, 8]. With AP2 being involved inneurotransmission and the trafficking of many other cell membrane proteins for signal transduction through clathrin-mediated endocytosis [15], it is logical to see patients with FHH-3 having cognitive and neurodevelopmental impairment.

In our report, the proband with *AP2S1* gene mutation in the Arg15His residue has ADHD with developmental and speech delay. Hence, our report adds to the existing hypothesis that the neurodevelopmental, cognitive, and psychiatric disorders seen in patients with FHH-3 might well be attributed to the *AP2S1* mutations. Further studies and reports are required to confirm this possible association.

## 4. Conclusion

The biochemical and clinical features noticed in our patient with FHH-3, in the form of hypercalcemia, hypocalciuria, and hypermagnesemia, and the presence of cognitive or psychiatric disabilities could be used to differentiate FHH-3 from FHH-1. Notably, a prudent counseling and follow-up can be offered in view of possible neurodevelopmental and cognitive impairment associated with FHH-3. A regular follow-up of these patients in an outpatient clinic with endocrinology and neurodevelopmental facilities is justifiable.

## Figures and Tables

**Table 1:** Summary of biochemical results.

	Patient (9 months)	Mother (34 years)
25-hydroxyvitamin D	23.3 ng/ml (normal)	5.2 ng/ml (low)
Calcium	11.2 mg/dl–10.9 mg/dl (8.8 mg/dl–10.8 mg/dl) (high)	10.6 mg/dl (8.9 mg/dl–10.2 mg/dl) (high)
Phosphate	4.9 mg/dl (4.9 mg/dl–7.9 mg/dl) (normal)	2.6 mg/dl (2.7 mg/dl–4.5 mg/dl) (low normal)
Magnesium	2.50 mg/dl (1.7 mg/dl–2.4 mg/dl) (high normal)	2.44 mg/dl (1.7 mg/dl–2.55 mg/dl)
ALP	313 u/l (<281) (high)	54 u/l (35–104)
PTH	8.6 pg/ml (6.2–29.0) (normal)	10 pg/ml (normal)
Calcium, random urine	3.7 mg/dl	17.2 mg/dl
Creatinine, random urine	17.4 mg/dl	304.4 mg/dl
Urinary calcium/creatinine ratio	0.21 mg/mg (low)	0.05 mg/mg (low)

ALP, alkaline phosphatase; PTH, parathyroid hormone.
